# A calibrated chronology of biochemistry reveals a stem line of descent responsible for planetary biodiversity

**DOI:** 10.3389/fgene.2014.00306

**Published:** 2014-09-11

**Authors:** Gustavo Caetano-Anollés, Jay E. Mittenthal, Derek Caetano-Anollés, Kyung Mo Kim

**Affiliations:** ^1^Evolutionary Bioinformatics Laboratory, Department of Crop Sciences, University of IllinoisUrbana, IL, USA; ^2^Department of Cell and Developmental Biology, University of IllinoisUrbana, IL, USA; ^3^Microbial Resource Center, Korea Research Institute of Bioscience and BiotechnologyDaejeon, South Korea

**Keywords:** structure, phylogenetic analysis, molecular clock, protein folds, protein evolution

## Abstract

Time-calibrated phylogenomic trees of protein domain structure produce powerful chronologies describing the evolution of biochemistry and life. These timetrees are built from a genomic census of millions of encoded proteins using models of nested accumulation of molecules in evolving proteomes. Here we show that a primordial stem line of descent, a propagating series of pluripotent cellular entities, populates the deeper branches of the timetrees. The stem line produced for the first time cellular grades ~2.9 billion years (Gy)-ago, which slowly turned into lineages of superkingdom Archaea. Prompted by the rise of planetary oxygen and aerobic metabolism, the stem line also produced bacterial and eukaryal lineages. Superkingdom-specific domain repertoires emerged ~2.1 Gy-ago delimiting fully diversified Bacteria. Repertoires specific to Eukarya and Archaea appeared 300 millions years later. Results reconcile reductive evolutionary processes leading to the early emergence of Archaea to superkingdom-specific innovations compatible with a tree of life rooted in Bacteria.

A stem line of descent is a primordial propagating series of pluripotent cellular entities. It is believed that such a remarkable backbone of the cellular world resulted in sequential cellular lineage spin-offs (Wang et al., 2007), very much as modern differentiated cell types arise from less differentiated embryonic or adult stem cells. The axioms of evolution supporting the genealogy of life seeded by the stem line demand that tree-like ensembles of lineages nested within each other populate the biological world (Wiley, 1975). The molecular, cellular, and organismal entities (taxa) that unfold in the nested lineages are the subject of gradual change, fulfilling spatiotemporal continuity and benefitting from non-vertical transfer of information. Taxa also retain features (characters) that are refractory to change and are preserved through genealogical descent. These characters allow dissection of the history of life and the generation of evolutionary chronologies embodied in time-calibrated phylogenetic trees (timetrees). As we will show, characters of this type, such as the highly conserved atomic structure of macromolecules (Caetano-Anolles et al., 2009), can also inform about the ancient history of the stem line and its makeup.

When taxa are organisms and characters are organismal component parts, trees represent explicit statements of organismal history. Once crucial divergences are time-calibrated with molecular, fossil and other evidence, the nested hierarchies represent “timetrees of life” (ToLs) (Donoghue and Benton, 2007; Laurin, 2012). The leaves of these traditional trees depict present-day organisms (sometimes extinct taxa representing species, genera, or families) and their evolution is viewed through the lens of the conserved features that are studied; for example, a tree of α-proteobacteria can be viewed through evolution of their ribosomal RNA molecules. Conversely, when taxa are the component parts themselves, the nested hierarchies describe a different kind of timetree that explores the chronology of component innovation of the organismal system. In particular, “timetrees of domains” (ToDs) are powerful trees that harbor leaves representing protein domain structures, their evolution viewed through the lens of their genomic abundance (Wang et al., 2011). Here we show that these time-calibrated trees uncover remarkable patterns of origin and diversification of biochemistry, striking evolutionary patterns supporting the ancestral stem line, and the time of emergence of planetary biodiversity.

## Phylogenomic retrodiction uncovers biochemical history

Biological “modules” that are sufficiently conserved are particularly useful characters for phylogenetic analysis. Modules are sets of component parts that interact more tightly with each other than with other parts of the system. They can be molecular (e.g., protein domains; Murzin et al., 1995) or of other kinds (e.g., developmental; Laurin, 2014). These cohesive units have the ability to diversify in the many different contexts of the cell, adding to biodiversity (Mittenthal et al., 2012). The number of module types increases in evolution; their growing numbers also distribute in nested manner in the nested lineages of the trees. Useful molecular modules include amino acid monomers in protein chains, sets of stabilizing H-bonding interactions in RNA molecules, structural motifs in folded proteins, or protein domain structures in proteomes. Since modules can be viewed as determinants of levels of biological organization, timetrees of modules that are part of the molecular and cellular makeup of the cells can provide useful information about biochemical history.

Evolved macromolecules are generally endowed with a finite conformational ensemble of 3-dimensional atomic structures (e.g., Fontana, 2002; Schuster, 2010). These folding conformations are highly dynamic and materialize for significant periods of time, enough to hold molecular functions that are advantageous to the cells. Consequently, their ensembles represent “living fossils” that have retained considerable history of their molecular past (Caetano-Anolles et al., 2009). They can be mined with modern bioinformatics tools of phylogenetic reconstruction.

**Figure 1:**
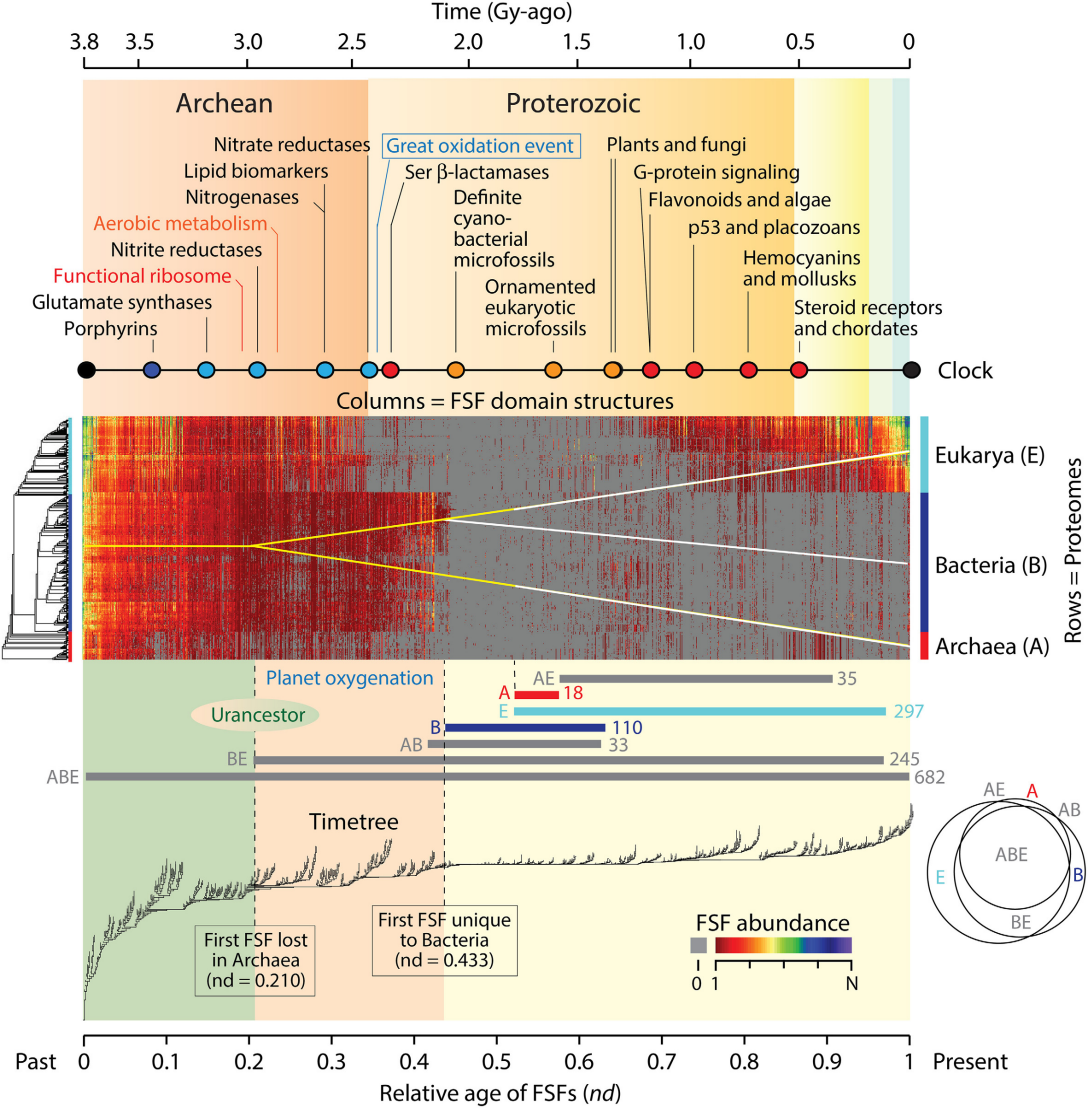
**A timetree of protein domain structures describes molecular history within the context of the geological record**. A census of protein domain structure in the proteomes of 420 free-living organisms representing the three superkingdoms of life was conducted at the FSF level of structural abstraction in SCOP (Kim and Caetano-Anollés, 2011). The color array of the evolutionary heat map in the center describes the distribution of genomic abundances of 1420 FSFs in the 420 organisms that were surveyed. Gray cells imply an abundance of 0 (the absence of the domain structure altogether). Red-to-blue hues represent increasing abundance levels, from 1 to *N* = 15,112 counts of a same FSF structure. Abundance values in the array were coded as discrete phylogenetic characters using an alphanumeric scheme 0–9 and A–N and arranged in transposable data matrices for phylogenetic analysis. Characters transform according to linearly ordered and reversible pathways. Maximum parsimony was used as the optimality criteria to generate a ToL (left of matrix) and ToD (below matrix) using a combined parsimony ratchet and iterative search approach. These trees were used to order rows and columns in the heat map matrix. The ages of FSFs are time-calibrated with a global molecular clock of fold structures that spans 3.8 billion years (Gy) of history and associates diagnostic domain structures with multiple geological ages derived from the study of fossils and microfossils, geochemical, biochemical, and biomarker data (colored circles: red, biochemistries and lineages; orange, organismal diversification; blue, nitrogen assimilation and other biomarkers; black, boundary events). Interpolations of crucial biochemical developments are indicated in the timeline (Kim and Caetano-Anollés, 2011). Below the heat map are evolutionary mappings of FSF sets belonging to Venn distribution groups of domains unique (A, B, E), shared (BE, AB and AE) or ubiquitous (ABC) among superkingdoms. The Venn diagram shows a significant number of shared FSFs. A tree of superkingdoms inferred from Venn group appearance in the timeline is overlapped onto the heat matrix, and depicts a possible stem-line of descent in yellow. We note that the timetree and molecular timelines that are shown benefit from standard molecular evolutionary techniques (e.g., phylogenies of sequences, physiologies, and morphology), inorganic and organic geochemistry (e.g., distributions of trace elements in shales or banded iron formations or concentrations of organic compounds like steroids that are diagnostic of certain taxonomies), micropaleontology and paleontology (the distribution of physical fossils, with morphology providing evidence for the presence of specific organisms), and other sources of history.

We have shown that the natural history of protein domain structures and their associated functions can be directly inferred from ToDs, comb-like trees that harbor domains as taxa (Caetano-Anollés and Caetano-Anollés, 2003). ToDs are built from a genomic census of protein domain structure (suitably defined) in the proteomes of thousands of genomes using the standard tools of cladistics analysis. Domains are the main structural, functional and evolutionary modules of proteins that are highly conserved and are recurrently arranged in multidomain proteins (Wang and Caetano-Anollés, 2009). The genomic census of folded structures can be defined using the different levels of structural abstraction of the accepted protein domain classification gold standards, the SCOP (Murzin et al., 1995) or CATH (Orengo et al., 1997) databases. Figure 1 shows a data matrix (array) of domain abundance in the proteomes of 420 free-living organisms belonging to the three superkingdoms of cellular life, Archaea, Bacteria, and Eukarya. The analysis excludes the history of viruses, which can be found elsewhere (Nasir et al., 2012). The phylogenetic matrix was visualized as a heat map, a graphical representation of the data in which values of domain abundance are represented with colors. Domains in the genomic census were defined at the fold superfamily (FSF) level of the SCOP hierarchical classification. FSFs unify a diversity of protein sequences that share a 3-dimensional fold structure, related molecular functions and a common evolutionary origin. In the data matrix, the proteomes of organisms (rows of the matrix) were ordered according to a rooted and ladderized ToL. Similarly, domains in proteomes (columns) were ordered according to the relative ages of FSFs (*nd*), which were derived directly from a ToD. The rooted ToL and ToD trees are shown to the left and below the heat map and were built using maximum parsimony from the data matrix and its transposed derivative, respectively.

We note that three properties enable timetree retrodictions from domain abundance data (Figure 1): (i) *The trees are rooted:* Rooted ToDs are built by using a process model that considers that the most abundant and widely distributed domain structures are of ancient origin (Caetano-Anollés and Caetano-Anollés, 2003). The model confers polarity (distinction between ancestral and derived states) to a transformation series of ordered multi-state phylogenetic characters that describe increases and decreases of FSF abundances in proteomes. This polarization roots the trees without invoking outgroup taxa or other external assumptions and can be validated by a number of criteria (Kim et al., 2013). It also complies with Weston's generality criterion (Weston, 1994), which is supported by homology in nested patterns and additive phylogenetic change and roots the trees with the minimum number of assumptions. Using the Lundberg method, the root is identified by attaching a hypothetical ancestor that is defined by the polarization model to an optimal unrooted tree in a most-parsimonious manner (Lundberg, 1972). (ii) *Chronologies are inferred directly from the trees:* Chronologies cannot be inferred directly from rooted trees that tend to follow stochastic or null branching processes of change, i.e., that are relatively well balanced. In these cases, time calibrations for origins of clades are achieved for example by the use of fossil data in ToLs. In contrast, when rooted trees follow semi-punctuated evolutionary processes responsible for accelerated change during divergence (Venditti and Pagel, 2010), they are highly unbalanced and pectinate in appearance. This is the case of ToDs, in which splitting of lineages depends on an evolving “heritable” trait (Heard, 1996), the gradual accumulation of structural variants of domains in lineages and the semipunctuated discovery of new domain structures. Chronologies can be inferred directly from these imbalanced trees by calculating a “node distance” (*nd*), the relative number of internal nodes from the root to a leaf of the tree. (iii) *Phylogenetic statements are linked to the geological record through time calibration points:* A global molecular clock of domain structures establishes a significant linear relationship between the age of domains and the geological record (Wang et al., 2011). Thus, FSF domain structures diagnostic of biomarkers and geomarkers provide the time calibration points.

The time of first appearance of a domain structure at FSF level in the chronology records the time of the origin of that FSF. Consequently, the chronology of FSFs should be viewed as a timeline of molecular innovation portraying the gradual rise of modern biochemistry (Caetano-Anolles et al., 2009). For example, we have used chronologies of these kinds to trace the origin and evolution of metabolic networks (Caetano-Anollés et al., 2007; Kim et al., 2012; Caetano-Anollés and Caetano-Anollés, 2013), study the rise of translation and the genetic code (Caetano-Anollés et al., 2011, 2012, 2013), uncover the coevolutionary history of the ribosome (Harish and Caetano-Anollés, 2012), explore the evolution of metallomes and biological metal utilization (Dupont et al., 2010), unfold the natural history of biocatalytic mechanisms (Nath et al., 2014), study the evolutionary dynamics of gain and loss of domains (Nasir et al., 2014), and determine the makeup of the common ancestor of life (Kim and Caetano-Anollés, 2011).

## Patterns of domain abundance reveal a megaorganismic stem line of descent

The heat maps of Figure 1 overlap domain abundance (and occurrence) on the data matrix used for timetree generation. They describe the reuse and spread of molecular innovations in the proteomes of the modern protein world. Older FSFs are expected to be more abundant and widely distributed (Wang et al., 2007, 2011). Indeed, the most abundant and widely spread FSFs that are universally distributed appear at the base of the chronology, within the first 0.1 Gy of protein history. These structures are part of an initial and universal core responsible for the primordial metabolic, structural, and cellular functions (Danchin et al., 2007; Wang et al., 2007), which we propose represents the most ancient repertoire of the stem line that gave rise to the molecular and organismal biodiversity of the planet. This stem line was probably embodied in a megaorganism, in the sense of a modern fusion-driven syncytium and/or a fission-driven coenocyte (such as the plasmodial slime molds; Egel, 2012), which preceded modern organisms and lineages. The entity probably resembled the multiphenotypical precells of Kandler (1994) that later seeded the concept of a communal ancestor (Woese, 2002). The FSF repertoire was most likely quasispecies-like and fluid, i.e., a cloud of genotypes and phenotypes changing at high rate (Seufferheld and Caetano-Anollés, 2013). It was cellular and harbored a molecular makeup that was in part associated with archaic membranes. Iterative phylogenetic character state reconstruction of the proteome of the urancestral line suggests it contained 303–507 domains apportioned into 70–152 FSFs at the time of the appearance of first lineages (Kim and Caetano-Anollés, 2011). Remarkably, conical laminated morphologies of probable biological origin (stromatolites) have been identified in the 3.4 Gy-old Strelley Pool formation of Western Australia (Allwood et al., 2006) and may contain microfossils of sulfur-metabolizing cells (Wacey et al., 2011). Our analysis now provides molecular backing to the biogenic interpretation of these rock microstructures.

While the proteomic repertoire of the stem line was 3–5 times smaller than that of standard free-living organisms, its makeup continued to expand. As time progressed in the timeline and new FSFs were uncovered, the differential accumulation of FSFs in proteomes makes evident the evolutionary expansion/reduction of proteome repertoires. It shows that a number of FSFs were gradually lost (or never gained) in archaeal organisms. These early diversification patterns suggest the emergence of archaeal grades by reductive evolution and the very early rise of Archaea as the first superkingdom of diversified life (Wang et al., 2007; Caetano-Anollés et al., 2014). Grades (*sensu* Huxley, 1958) are variants of the stem line in active transition but unified by the same level of physiological complexity stemming from the FSF repertoire (Caetano-Anollés et al., 2014). We note that complete loss of an FSF in a proteome is most likely to occur soon after the origin of the FSF, when there are few copies of it encoded in the genome. Since genomic abundances of each FSF increase in evolution as genes duplicate and diverge or are created *de novo*, it is increasingly less likely for domain loss to occur later on in evolution. This is especially so if loss occurs in all proteomes of a superkingdom, such as the first FSF that was lost completely in all lineages of Archaea ~2.9 Gy ago. It is also noteworthy that when domain abundances in FSFs increase, the probability of recruitment of FSF variants to perform different functions in different cellular contexts also increases. This fosters domain innovation, domain interactions, multidomain proteins, and the formation of cellular complexes. Thus, the age of an FSF is just the lower bound on its use as a module—maybe a bound unlikely to be attained—since modularity unfolds gradually in evolution (e.g., domains as recombining units; Wang and Caetano-Anollés, 2009).

## The rise of planetary biodiversity began ~2.9 Gy ago and materialized ~2.1 Gy ago following the “crystallization” of modern cellular modules

The same reductive evolutionary trend of differential accumulation of FSFs different from the stem line that starts in Archaea ~2.9 Gy ago is seen for the first time ~2.8 Gy ago in Bacteria and Eukarya (their common ancestral line), revealing further weakening of the stem line in favor of grades (Figure 1). This becomes especially evident 2.45 Gy ago, the time of the Great Oxygenation Event (GOE) of the planet (Sessions et al., 2009). Abundances of new FSFs in Eukarya drop precipitously at this time in the heat map, while those in Bacteria were maintained for an additional 3–4 million years (Figure 1). The fact that these sharp reductive changes manifest with prior and time-localized increases of FSF abundances in Eukarya 2.6–2.9 Gy ago and in Bacteria ~2.4 Gy ago is remarkable. It suggests a complex dynamics of domain growth in FSFs unfolding close to the transition point (threshold or crystallization; *sensu* Woese, 2002) between the megaorganismic precells and grades and the rise of modern cells and lineages. This transition is marked by the appearance of the first superkingdom-specific FSFs in Bacteria ~2.1 Gy ago and coincides with the age of the oldest unambiguous microfossils of cyanobacteria (matching the genus *Archaeoellipsoides*) that are preserved in 1.5–2.1 Gy old cherts throughout the world (Tomitani et al., 2006). We note that the appearance of these FSFs should be regarded as unequivocal. Their origin cannot result from transfer or massive loss of structures in other superkingdoms.

## Likely culprits of planetary biodiversity: oxygen and aerobic metabolism

Timetrees suggest that aerobic metabolism and planetary oxygenation appeared concurrently ~2.9 Gy ago (Wang et al., 2011), ~400 million years before the GOE, and that the Mn catalase enzyme was the ultimate culprit of oxygen production (Kim et al., 2012). Chemoinformatic dissection of the chemical space of metabolites showed that planetary oxygen shaped the chemical makeup of metabolites in aerobic metabolic networks, expanding the structural and chemical space with 130 new molecular scaffolds of the 335 total set (Jiang et al., 2012). Oxygen also made metabolites more rigid and hydrophobic, when these were analyzed with a range of chemical property descriptors (e.g., rotable bond counts, polar molecular volume, hydrophobic fragment count). These properties prompted cellular diversity. For example, the polar makeup of steroids influences endo/exocytosis and transmembrane trafficking, poising the transition to multicellularity (Summons et al., 2006). It is also likely that planetary oxygenation resulted in extinction of a significant part of anaerobic biodiversity, enabling novel organismal radiations and unfolding adaptations in response to the new noxious environments, which are currently reflected in links between metabolism and ontogenesis (Herkovits, 2006). We therefore propose that both oxygen and aerobic metabolism enhanced the complexity and diversity of cellular organization.

## Conclusion

Phylogenomics makes explicit the differential evolutionary accumulation of domain structures in evolving proteomes. Evolutionary patterns suggest that the primordial stem line produced cellular grades 2.9 Gy ago, which slowly turned into archaeal and eukaryal lineages and into bacterial lineages at more accelerated pace. This differential behavior reconciles the early origin of Archaea inferred from molecular structures and functions with the canonical rooting of the ToL sometimes revealed by standard sequence analysis. The important realization that supports these findings is the continuous growth of the protein world and the fact that the complete loss of a domain structure in a proteome occurs more likely soon after its origin in the timeline, at a time when there are few domain copies encoded in the genome and recruitment is incipient.

### Conflict of interest statement

The authors declare that the research was conducted in the absence of any commercial or financial relationships that could be construed as a potential conflict of interest.
